# A Bias Network Approach (BNA) to Encourage Ethical Reflection Among AI Developers

**DOI:** 10.1007/s11948-024-00526-9

**Published:** 2024-12-17

**Authors:** Gabriela Arriagada-Bruneau, Claudia López, Alexandra Davidoff

**Affiliations:** 1 Instituto de Éticas Aplicadas, Instituto de Ingeniería Matemática y Computacional, Pontificia Universidad Católica de Chile, Avenida Vicuña Mackenna, 4860 Santiago, Chile; 2 Departamento de Informática, Universidad Técnica Federico Santa María, Avenida España, 1680 Valparaíso, Chile; 3 Sociology of Childhood and Children’s Rights, Social Research Institute, UCL. 20 Bedford Way, London, UK; 4 Nucleo Futures of Artificial Intelligence Research (FAIR), Santiago, Chile; 5 Centro Nacional de Inteligencia Artificial (CENIA), Santiago, Chile

**Keywords:** AI bias, AI ethics, Sociotechnical, Professional bias, Decision-making

## Abstract

We introduce the Bias Network Approach (BNA) as a sociotechnical method for AI developers to identify, map, and relate biases across the AI development process. This approach addresses the limitations of what we call the "isolationist approach to AI bias," a trend in AI literature where biases are seen as separate occurrences linked to specific stages in an AI pipeline. Dealing with these multiple biases can trigger a sense of excessive overload in managing each potential bias individually or promote the adoption of an uncritical approach to understanding the influence of biases in developers’ decision-making. The BNA fosters dialogue and a critical stance among developers, guided by external experts, using graphical representations to depict biased connections. To test the BNA, we conducted a pilot case study on the "waiting list” project, involving a small AI developer team creating a healthcare waiting list NPL model in Chile. The analysis showed promising findings: (i) the BNA aids in visualizing interconnected biases and their impacts, facilitating ethical reflection in a more accessible way; (ii) it promotes transparency in decision-making throughout AI development; and (iii) more focus is necessary on professional biases and material limitations as sources of bias in AI development.

## Introduction

Non-discrimination principles in AI promote bias prevention or mitigation as primary tactics to achieve fairness (Fjeld et al., 2020; Jobin et al., 2019), successfully raising awareness about hidden AI biases and creating tools for harm prevention. Yet, these strategies are commonly viewed as isolated remedies applied at specific stages within an AI pipeline, neglecting broader societal influences and the comprehensive impact of bias sources on the entire AI process.

This is what we call the “isolationist approach to AI bias.” We think it comes from two trends: (1) a technocentric view that focuses on creating technical solutions that are tailored to specific problems, leading to many new but possibly unconnected solutions; and (2) a reliance on technocentric solutions as the primary way to deal with sociotechnical issues related to bias. This poses a substantial challenge for AI developers as they navigate a landscape fraught with multiple biases demanding attention, particularly in critical sectors such as healthcare or education. Unlike specialized researchers concentrating on individual biases for building new techniques, AI developers grapple with deploying AI ethically across diverse applications, facing practical complexities. Identifying and addressing multiple biases can be daunting, particularly for smaller AI teams lacking ethical training.

To counter the isolationist view, we will propose a sociotechnical method called “the bias network approach” (BNA) to deal with biases as an interconnected network instead of isolated elements. Even though there are different ways to deal with biases, such as oversampling techniques (Buolamwini & Gebru, 2018; Zhou et al., 2023), most of them only affect one step in an AI pipeline, like data collection, and do not take into account how biases might affect later stages of development. Hence, our objective is to highlight the links between biases and human input, bringing these interactions to the forefront of AI developers' ethical deliberation. As a result, our method aspires to improve bias evaluations, comprehend potential risks, and foster ethical awareness within the AI development community.

Our proposal will be grounded in sociotechnical perspectives, emphasizing the significance of considering broader contexts when analyzing ethical issues such as bias. Echoing West et al. (2019), we recognize that as AI’s ethical implications become more prominent, our examination must extend beyond technical biases to include the influence of developmental environments. This comprehensive view acknowledges that biases are not just technology artifacts but also products of cultural, institutional, and human factors.

Our paper consists of three main sections. In “Related Work: Isolationist Perspectives to Address Biases in AI” section, we describe and critique the prevalent approach to addressing AI bias, which we call the “isolationist approach” where technical, societal, and human biases are often linked to specific steps of the AI development process, neglecting how they may stem from shared causes or contribute jointly to problematic outcomes. In “Methodology” section, we outline the fundamental elements of the BNA. We also introduce the graphical representation for mapping bias sources and impacts and the proposed conversational method tailored for its application. In “Results” section, we present a case study demonstrating the BNA’s application through a retrospective analysis of the “waiting list” project in Chile’s healthcare domain. Our qualitative analysis involved open coding and thematic analysis. Finally, we present three key findings resulting from implementing the BNA:

(i) Highlighting material limitations and external decisions as significant bias sources.
(ii) Acknowledging the importance of professional biases identified using our approach.
(iii) Demonstrating the benefits of employing the BNA in the revision stage, showing its potential to facilitate ethical reflection and improve transparency.

Our approach shows promise as a method for AI developers, aiding in the integration of ethical awareness into AI professional practice and acknowledging limitations.

## Related Work: Isolationist Perspectives to Address Biases in AI

Prior research often classifies AI biases into societal, technical, and cognitive (Ferrara, 2023; Ntoutsi et al., 2020; Rajpurkar et al., 2022; Roselli et al., 2019; Soleimani et al., 2022; Zajko, 2021). The groups reflect distinctions associated with the context where biases emerge.

- Societal biases involve complex interplays of historical, institutional, and social forces, not necessarily explicit prejudice but reflecting prevailing behaviors and structural inequities.
- Technical biases encompass representation disparities and systematic statistical errors, leading to discernible partiality or discriminatory effects.
- Cognitive biases entail systematic human errors, like implicit biases and heuristics, impacting decisions throughout AI development and affecting human decisions and behaviors during AI use.

A prevailing trend in bias identification and mitigation strategies is what we call an **isolationist approach**. This approach in AI bias literature associates each bias type primarily with specific stages in the AI process, like data collection or model development.

Most biases in prior literature are linked to one or a few steps of the AI process. We use a pipeline representation of the AI development process, following prior work (e.g. Barocas & Selbst, 2016; Suresh & Guttag, 2021); however, we acknowledge the limitations of this representation as it hides the complex and iterative nature of the AI development process (Stevens, 2013). By positioning biases in the AI pipeline, we highlight the segmented treatment of biases across the development steps—from problem definition and data management to model development, deployment, and feedback (Table 1).

**Fig. 1:**
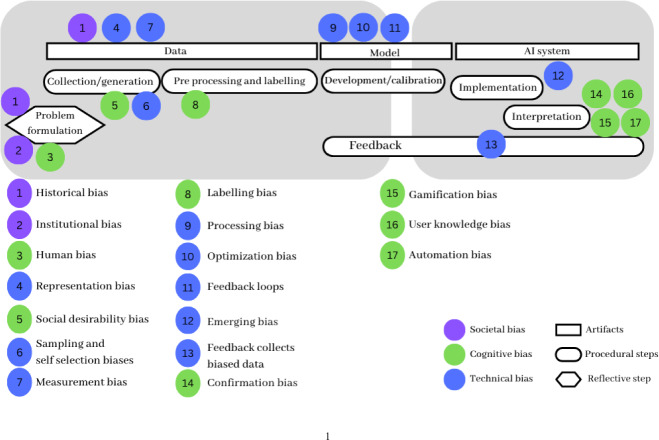
Biases as often mentioned in the literature, placed in different stages of the AI pipeline. (This basic pipeline figure has been slightly edited and used in our prior work [Omitted for blind review])

Figure 1 outlines biases identified in the literature, the AI development steps they relate to, and their corresponding categories (see more details in Annex, Table 2). While these bias categories encompass broader segments of the AI pipeline, they primarily concentrate on subsets within it. For example, **societal biases** are often mentioned at the beginning of the AI pipeline. They extend from problem formulation to data-related steps. For example, institutional (Ntoutsi et al., 2020) and historical biases (Suresh & Guttag, 2021) influence problem formulation. Historical biases are even more often associated with data issues, which can encode patterns of structural discrimination over time (Char et al., 2020; Dobbe et al., 2018; Mehrabi et al., 2021).

**Cognitive biases** are prominent in the initial and final stages of the pipeline. Social desirability might affect initial data generation (Olteanu et al., 2019), while pre-existing beliefs and biases could influence processing and labeling (Smith & Rustagi, 2020). Additionally, these biases arise when interpreting AI results, as users may introduce gamification bias to manipulate models for preferred outcomes (Richardson & Gilbert, 2021).

**Technical biases** are concentrated in the middle segment of the pipeline. Several appear within data collection, encompassing sampling, measurement, and selection biases (Akter et al., 2021; Cramer et al., 2018; Srinivasan & Chander, 2021). Processing biases hinder model learning and generalization, including aggregation biases (Barocas & Selbst, 2016; Mitchell et al., 2021). Emergent bias arises when there are disparities between algorithm design and its use, especially in contexts differing from the model's initial development (Draude et al., 2019).

Sources of bias are also identified at each step of the AI pipeline (see Annex, Table 3). During problem formulation, biases can stem from discrepancies between the problem and the affected population (Mitchell et al., 2021) or a lack of diverse perspectives in problem framing (Baker & Hawn, 2021). Subsequently, issues may arise from variable selection (Fazelpour & Danks, 2021). Data-related challenges include insufficient variable granularity (Feuerriegel et al., 2020; Kizilcec & Lee, 2022), incomplete or missing data (Parikh et al., 2019), and poor data notation (Paullada et al., 2021).

Problematic data variable relationships like spurious correlations (Parikh et al., 2019) and proxy variables (Feuerruegel et al., 2020) contribute to biases. Model-related biases can result from improper variable use and skewed data (Sangokoya, 2020), biased processing, and feedback loops (Akter et al., 2021). Biased models might also stem from inadequate performance metrics (Suresh & Guttag, 2021) and unsuitable benchmarks (Srinivasan & Chander, 2021). Other challenges include oversimplification (Barocas & Selbst, 2016), and overlooking crucial variables (Fazelpour & Danks, 2021), especially when fairness is not integrated into evaluation criteria (Mitchell et al., 2021). Implementation biases can result from inadequate preventive measures and monitoring (Fazelpour & Danks, 2021).

Numerous biases in AI stem from overlooked decisions or inherent flaws in the AI artifacts. To achieve bias-aware development, addressing these sources and their potential biases comprehensively is essential. Each bias might require specific mitigation strategies. which, if seen as separate issues, can overwhelm teams, hindering progress toward bias-aware AI. Our approach will seek to counter the isolationist view by highlighting connections among biases, their origins, and their ramifications. By identifying more impactful sources and biases, it aims to improve ethical awareness and decision-making for AI teams.

### A Bias Network Approach (BNA) to Guide Ethical Reflection

The isolationist approach we identified has at least three limitations: (i) It limits consideration of contextual concerns during decision-making processes; (ii) It leads to a disconnected understanding of biases and mitigation strategies that must be addressed, potentially impeding AI teams from effectively reflecting ethically about their decision-making; and (iii) It may lead to recurrent instances of risk because if biases are seen as individual instances requiring mitigation, the same bias or an effect stemming from a prior bias could resurface in subsequent development stages.

Our core proposal is based on sociotechnical approaches to AI ethics, which draw inspiration from systems theory to challenge the notion of technology as a detached entity within organizations. These approaches shed light on the intertwined nature of technological progress and societal frameworks, as pointed out by Niehaus and Wiesche (2021). Sociotechnical approaches serve as a lens to examine the interactions between users, designers, and developers with the tools and frameworks they engage with, all within the social context that shapes their development. Our proposal aligns closely with the concept of moral imagination, as it will be shown in the pilot case study. Lange et al. (2023) define moral imagination as:

the ability to (i) register that one’s perspective on a decision-making situation, including the available options and the normative factors relevant to adjudicating those options is limited; and to (ii) creatively imagine alternative perspectives that reveal new approaches to that situation or new considerations that bear on the competing approaches.” (p. 6)

In their proposed method, Lange et al. (2023) use this concept through a series of workshops with engineering teams, guiding participants to articulate their values, challenge initial assumptions, and explore ethical trade-offs.

Similarly, our proposal encourages stakeholders to envision technology, particularly AI, not as a neutral or isolated tool, but as part of a complex web of human values, assumptions, and social impacts. Thus, the BNA seeks to promote moral imagination by raising awareness that the ethical dimensions of technology emerge from the interconnectedness of social, technical, and moral domains in the context of dealing with biases in AI. It invites individuals to question and broaden their initial perspectives, thus achieving a more holistic view that triggers ethical foresight and anticipatory governance by exploring diverse scenarios, anticipating potential harms, and considering alternative paths.

The BNA is a framework designed to map and assess biases in AI by incorporating decision-making processes, material limitations, and external factors often neglected in traditional bias mitigation strategies. The goal is to track factors that impact the creation, promotion, or reinforcement of biases, allowing developers a more comprehensive assessment. Decision-making involves human-generated objectives, the identification of issues, assumptions, observations, principles, and overarching conclusions. It encompasses the participation of internal actors (such as developers and users) and external entities (like governments, data providers, and hosting institutions). These sources hold the potential to introduce biases rooted in societal or cognitive factors, which can significantly impact AI development by perpetuating or generating biases. Material limitations impose constraints on essential resources (like data and the workforce), hampering the progression of AI projects. These limitations often stem from external influences on developer teams, shaping their decision-making processes and options for mitigating biases.

The BNA involves both developer and prompter teams, with the latter facilitating a critical, non-prescriptive dialogue, supported by visual tools to map and illustrate bias interconnections. It is advised that the prompter team includes at least one member with technical AI training, alongside experts in philosophy, ethics, or social sciences with interdisciplinary experience. These roles, filled by internal or external experts, seek to foster reflective dialogue by presenting diverse perspectives. Such interactions aim to nurture the developer team's moral imagination, deepening their ethical awareness in their work.

**Fig. 2:**
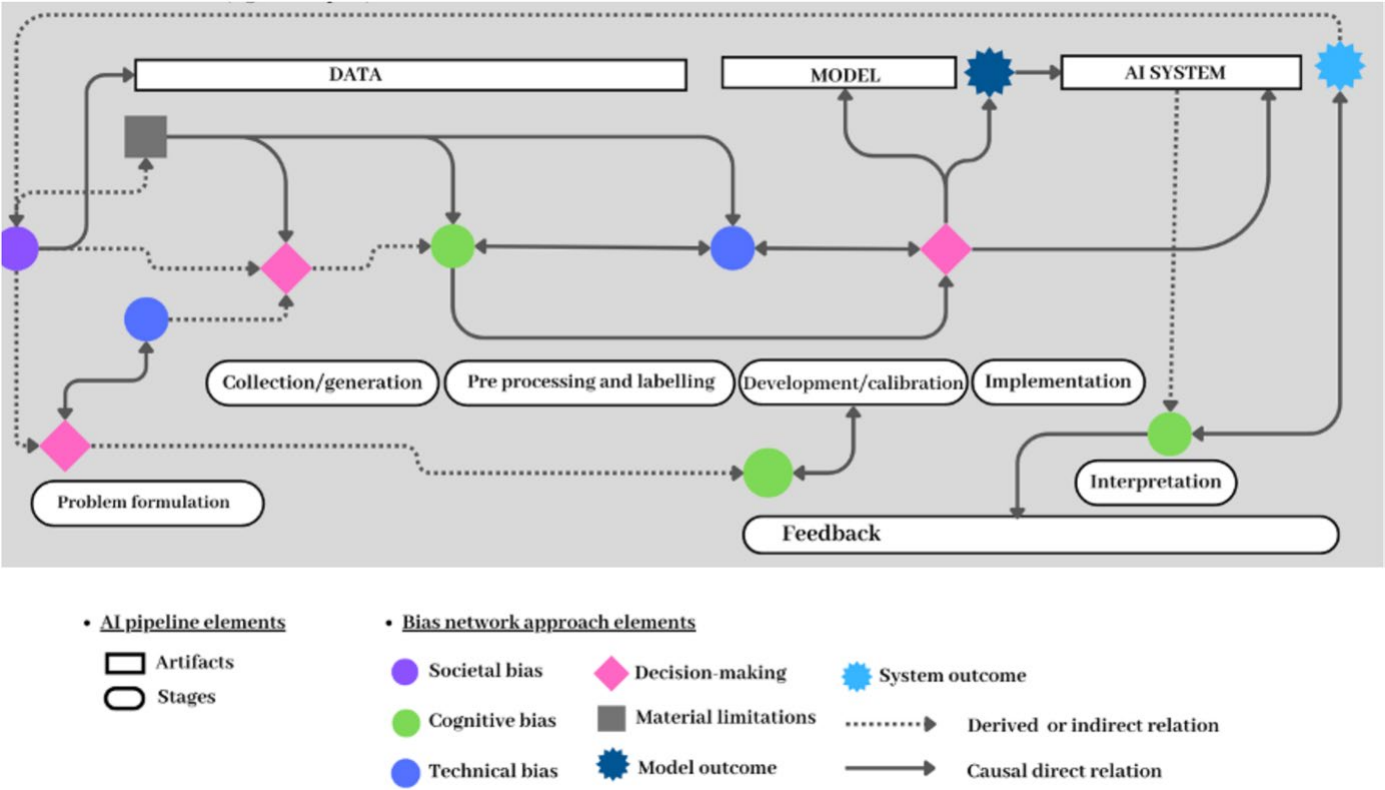
Network map to illustrate connections between biases, their sources (decision-making and material limitations), and outcomes (at model and system level)

To represent these interconnections visually, we crafted an illustrative network diagram (Fig. 2). To establish the basis for the bias network and prompt the developers to reflect, we used the adapted AI pipeline structure in Fig. 1 to make it more accessible for the developers. While the unidirectional “pipeline” metaphor is recognized for potentially oversimplifying the complex, iterative processes inherent in AI development (Stevens, 2013), we chose to use the AI pipeline representation to leverage its status as the prevailing perspective familiar to most developer teams (Beer, 2019) to facilitate and encourage critical reflection.

Furthermore, our approach distinguishes two distinct outcomes of biases and their causes: model outcomes in pre-deployment and system outcomes in post-deployment. Additionally, two types of relations connect these elements: direct or causal and indirect or derived. This classification acknowledges when biases might exert the most influence, potentially complicating the mitigation process.

### A Case Study to Assess the BNA

We conducted qualitative research to delve into participants’ subjective experience with the BNA, retrospectively assessing their decision-making process in the ‘waiting list’ project, which applied AI to process the healthcare waiting lists automatically in Chile. The case study aimed to gain insights from a team lacking formal ethics training but harboring concerns regarding ethical considerations in their research. This aspect fostered an open-minded approach during the case study.

Several factors guided our choice of the case study:

- High social impact: To employ sociotechnical contextualization for bias analysis, we selected a project with inherent ethical concerns due to its data or application context, specifically in healthcare.
- A small, predominantly engineer-based team: This choice targeted a team with limited ethical training and focused on a project with a core technical team making crucial methodological decisions.
- An existing model: While the BNA ideally starts from a project's inception, our exploratory pilot employed a retrospective analysis of a developed model. This was designed as a more manageable approach to gathering initial perspectives and insights. These insights would pave the way for testing the approach on different projects at various developmental stages in the future.

### Description of the Case Study’s AI Project

The team created an advanced natural language processing model that identifies essential information in medical and dental referrals for appointments in Chile’ public hospitals (Báez et al., 2022). The model identifies seven categories (e.g., disease, body part, medication, procedure) in the referrals’ text, scoring 80.27 for F1, surpassing the baseline neural model. These categorizations help reveal patterns, like diseases with more pending referrals to healthcare services, generating insights for public health decisions.

The Ministry of Health uses the waiting list as an official tool. The list includes referrals for diseases not covered by explicit health guarantees, which offer strict appointment timeframes with specialists. In contrast, those on the general waiting list face extended waits for specialist appointments. Chile's public hospital waiting list poses a critical issue impacting patient care, with an average wait surpassing 400 days in 2017 and over 1.5 million individuals awaiting referrals (Estay et al., 2017).

The team claims their work contributes to optimizing medical resource distribution and advancing epidemiological research (Báez et al., 2022). Their work started by gathering data to develop the model, requesting information from 29 healthcare services in Chile through the Transparency Law. They received positive responses from 23 services; the dataset comprised 5,157,902 referrals across 40 medical specializations and 11 dental specializations, with 88% focusing on medical records and 12% on dental records. Among 994,946 distinct diagnostic terms, they chose a subset of 107,235 terms exceeding 100 characters for annotation.

The team constructed a ground truth with 2067 dental and 2933 medical annotated referrals, showing an overrepresentation of dental cases. Dental referrals comprised roughly 41% of the ground truth, despite constituting only 12% of the total dataset (Báez et al., 2022). The team formulated a comprehensive codebook to classify seven distinct entity categories.

As of now, the model has not been implemented in the healthcare system. The team collaborates with the Ministry of Health to iterate and enhance the model and AI systems for clinical management decisions. As they continue to work towards integrating their findings into practical healthcare settings, they have agreed to use the BNA to reflect on areas for improvement and gain awareness for future evaluations and projects.

## Methodology

We used semi-structured interviews for each intervention (instances where the developer and prompter team interacted), as they provided a flexible yet systematic framework for analysis. In the initial two-hour session, we applied the BNA, using open-ended questions and prompts to elicit participants’ perspectives and stories behind their decision-making. This encouraged a discussion on the links between their choices, identified biases, and related sources. Our study underwent an ethical committee revision at the [omitted institution] and was approved under ID: 230,810,003.

We, the three authors—an ethicist, an informatics engineer, and a sociologist—interviewed three members of the waiting list project team: a researcher in applied mathematics and AI in healthcare, a computer science engineer, and a dentist with medical informatics expertise. Two of them later engaged in a 90-min follow-up session to discuss the BNA outcomes. Another team member, a biomedical engineer, did not participate in the interviews. The participants' diverse backgrounds were key to the study's findings.

Using Fig. 1 as a guide, we asked participants to think about how they made decisions at different stages, focusing on how they came up with problems and the next steps. We also asked them to think about and deal with common biases. Our questions were based on literature-identified biases to minimize our influence on the responses. Follow-up queries were directly related to the developers’ initial procedures and choices, allowing for deeper insight into their perspectives, such as “How did you formulate the problem, and how does it relate to the data collection decisions you made?" and “To what extent did you consider any biases we are showing you here? Or others that might come to mind,” and “Did any of your initial considerations change after data acquisition?”.

Interviews were audio-recorded, transcribed, and analyzed using open coding and thematic analysis, generating 46 codes representing biases and related factors. We closely examined each bias source, noting explicit interconnections mentioned by developers. We distilled seven key themes from this analysis, each of which we supported with concrete participant examples to highlight their importance. These will be detailed in the findings, depicted through an illustrative map that visually represents the relationships among various elements within the BNA.

## Results

We present two sets of findings: (1) Regarding the application of the BNA in the case study, these findings illustrate the potential of the networked approach in identifying influential sources of bias in AI projects, and (2) in relation to the developers’ views on the pros and cons of the BNA.

While discussing the findings, we identify participants as Developer A, Developer B, and Developer C, mainly to give the same importance to all their interventions (by not distinguishing a principal investigator, for example). The direct quotations by each developer have been slightly adapted in the Spanish–English translation.

### Findings About the ‘Waiting List Project’ as a Result of Applying the BNA

During the analysis, we observed that the thematic groups established connections among various sources of bias, linking decisions, influences, and limitations throughout the pipeline, i.e., there was an interconnection across steps. The subsequent sections emphasize the elements that had a more widespread influence on the project. These discoveries illustrate how the BNA effectively articulates the origins and effects of biases.

### The Decisive Role of Material Limitations and External Decisions as Bias Sources

**Fig. 3:**
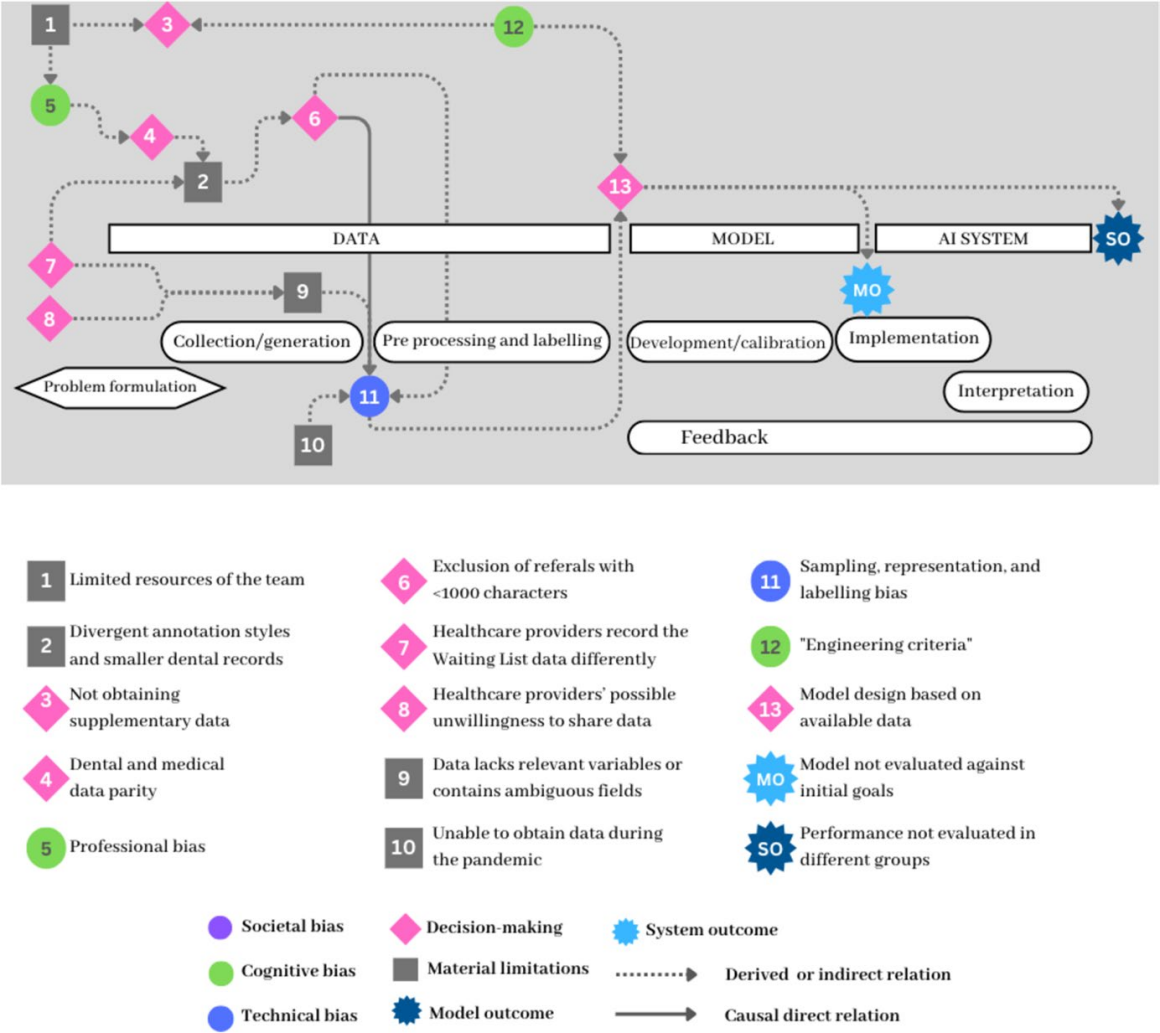
Network map showing the material limitations and decisions influencing model design, as identified by the AI developers

Figure 3 illustrates various issues associated with recognizing that several crucial material limitations and external decisions significantly influenced the emergence of sampling, representation, and labeling biases in the waiting list project (11). The team faced significant constraints due to its limited resources (1), inability to access data during the pandemic (10), and healthcare providers' unwillingness to share data (8).

Developer B: “We didn’t have data, and when I say we didn’t, I mean it. In the beginning, we did not have an agreement with the Ministry of Health; then we were in the middle of the pandemic, so nothing was available. So, what we did was ask for the data through the transparency law, but even then, the healthcare institutions were not forced to give us any data. There is an exemption that states that if it is too time-consuming, they can opt not to provide any information. So, we had to beg, like, say please, we want to help, so send us anything you have or anything you can.”

In this scenario, the waiting list team was just content with having data to work with. Consequently, during the interview, when we highlighted potential biases in data collection, one developer remarked that:

Developer C: “They [healthcare institutions] sent whatever they had. We had no clarity about the timeframe for the data. But yeah, there were important differences. If they had access to digital data, it would be easier or better structured; the thing was that there was no uniformity in how they recorded it. Some centers keep manual databases as a parallel tracking system, so we could not really know.”

This difficulty in obtaining data and variations in data type and quality (labeled as 2) led to additional complications. The developers noticed a bias in how they decided to set a threshold (labeled as 6) on the selected training data:

Developer B: “There was this criterion based on how people wrote a diagnosis. They all tend to be short, so we decided that we would use only the ones with 100 characters. We selected referrals like that, and those were the ones we finally annotated.”

Recognizing that the quality and style of records significantly vary across different specializations and healthcare providers (labeled as 4), the decision to only select records of a certain length played a pivotal role in shaping their final model. By excluding shorter records (more common in dental entries), they could have introduced a bias that may have impacted the model’s performance and the quality of its outputs, particularly in contexts where shorter records are prevalent. The team identified it as a potential bias and emphasized the interconnectedness of their own decisions with external limitations.

Developer A: “We decided to have dental and medical data parity, but we could be introducing a bias thereby overrepresenting dental data. Because we did have more medical data, there are also more of those in the system, so I don't know if this is a bias that comes up just now. [...]I also think there was an idea of using the limited human resources we had—having a dentist—and we kind of said, “We need to use that."

Developer C complements: “Yeah, actually, the difference between medical and dental data was big. There were 42 words on average used in medical diagnoses and only 28 in dental ones. I think we just put it all on a table, but we did not make any conscious decisions about it; it was not part of “building the model.” It was just data sampling, but there was a bias in that. We are just applying what we were able to gather without really thinking about how the data gathered by other professionals is affecting how we think about our model.”

**Fig. 4:**
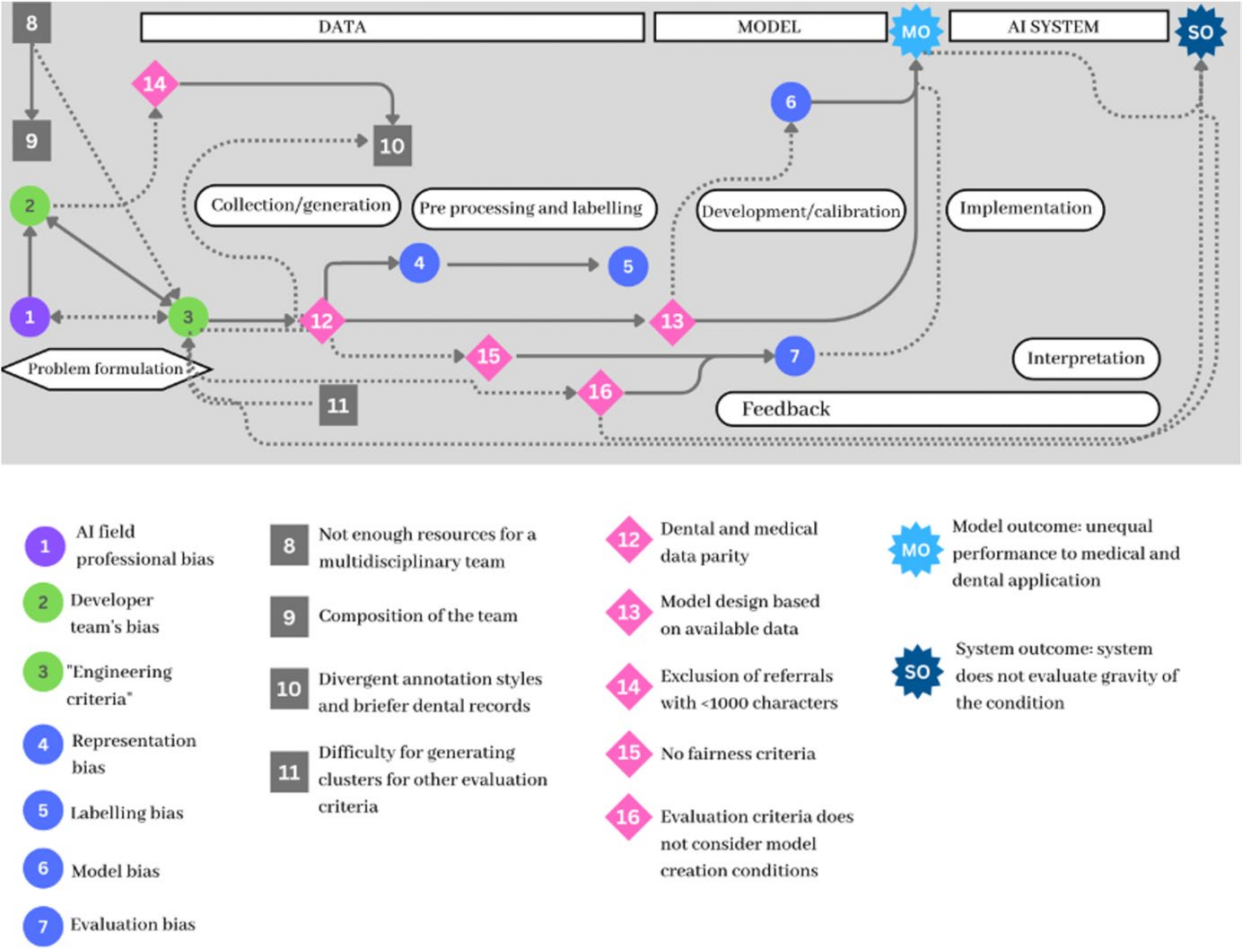
Network map showing the influence of professional bias as identified by the developers

In analyzing developers’ responses, we noted their awareness of conflicting interests among stakeholders affecting their project, particularly in data sharing by external institutions. This, along with the healthcare system’s structure, significantly influences bias emergence, as depicted in Fig. 4. Data intended for operational documentation, not AI training, may lack relevant fields and can be restricted or altered by external actors, affecting its completeness and accuracy. Moreover, disparities in data quantity and quality across medical specializations pose additional challenges, influenced by each field’s unique culture and appointment volumes. For instance, cultural perceptions in fields like gynecology affect data representativeness.

### The Pervasiveness of Professional Biases

The BNA indicated that professional bias significantly affects AI development, a factor often understated in AI bias literature. This bias, prevalent in the AI team's decision-making (referenced as label 1 in Fig. 4), reflects a preference for technical solutions rooted in the developers' engineering backgrounds. While not deliberately ignoring ethical issues, the team’s choices often missed crucial sociotechnical aspects due to a lack of awareness about the broader implications of their decisions.

In AI ethics, researchers criticize the reliance on technical solutions for ethical AI issues, known as technocentric solutionism (Peeters et al., 2021). Yet, there is a gap in understanding why developers gravitate towards this technocentric approach from a professional standpoint. As developer C noticed, it can be associated with a lack of ability to look outside their technical focus.

Developer C: “We were so focused on the technical side that we did not give ourselves any time to think about things around us or look at them from a distance, from the outside. That is why we did not have this type of discussion before.”

This phenomenon has been discussed in engineering ethics. Davis (1998) introduces the concept of “microscopic vision,” referring to the narrow focus and detailed problem-solving approach characteristic of engineers. Davis argues that engineers adopt a microscopic perspective when tackling technical challenges, as they are trained to break down complex problems into smaller, more manageable components. Developer B called it their “engineering criteria”:

Developer B: “I think there is a very strong bias present here, the fact that we all come from an engineering-related background. I mean, the only thing we truly cared about was getting the best performance—the F1 score. So, we looked at the literature to see models and metrics to do just that—our “engineering criteria.” But I never really stopped to think, How is that “best metric” going to help?”

Microscopic vision then refers to a selective focus on specific information deemed more valuable in a given situation. This heightened perception comes with the trade-off of potentially overlooking other essential aspects, such as social interactions or broader contexts. After identifying the professional bias (in Fig. 4), developers questioned their “microscopic vision”:

Developer B: “So maybe... we should also look at the public health structure and how the model influences what is done there. If we try to get out of our comfort zone to foresee these possibilities, maybe we can truly help people and create models that can be reproducible.”

Developer A: “I even question the fact that if we are all primarily engineers in a team, but we are working on medical stuff, then you have to make sure you assess the representativeness of that domain to identify deficiencies.”

Achieving a balanced perspective requires individuals to set aside their microscopic vision momentarily and consider the broader context to understand the overall situation more comprehensively. The team even considered further contextualization in relation not just to their design and testing process but also to implementation challenges (which are not yet finalized):

Developer B: “Like, I know our solution is not perfect, but it is better than what the Ministry has. So maybe having more consideration for their context can help us show them why they need this. Developer C gave some training to a few experts at the statistics department in the Ministry of Health. However, the rest of the people involved in implementing the model had no idea about the technical or other social benefits. We should have some sort of online feedback to report errors or worries. We did not consider that.”

Developer C: “I think that the big problem is the users. Like who is going to use the system and how. There is a bit of a blind spot about users, which in part was caused because we did not consider this side but also because there are structural limitations and bureaucracy to define who the final user will be inside the Ministry. We need to consider that limitation.”

A complementary concept to microscopic vision is “professional deformation.” Polyakova (2014) introduces this to talk about professionals in a particular discipline undergoing a cognitive transformation that makes them develop specific patterns of thinking and behavior strongly influenced by their professional training. This can instill a narrow perspective on how to approach interdisciplinary problems. In the case study, this was reflected in how they were discussing fairness, mainly through metrics and without much consideration for broader context:

Developer B: “Beyond performance metrics or accuracy adjustments, we did not think about “fairness” in general or in a broader sense.”

The developers identified their professional biases as major influences on their decision-making and problem-solving while creating their model. This led to a lesser focus on, or unawareness of, representativeness issues and societal biases, particularly in areas like medical data and the gynecological field.

Accordingly, we consider that professional bias should be more explicitly discussed in the AI bias literature as a type of bias that transcends the typical categories of societal, cognitive, or technical. Instead, it sheds light on the practices and methodological limitations AI developers acquire through their professional exercise.

### AI Developers’ Reflections on the BNA

#### The Role of Network Visualization and its Potential for Transparency Efforts

In the second interview, developers often reflected on material constraints and their decisions. They found value in visualizing these factors and the inherent connections in their analysis. Adopting a sociotechnical lens, they realized how external societal influences shaped their approach to model building and the significance they placed on technical choices, uncovering previously overlooked or unrecognized biases:

Developer B: “It is pretty overwhelming all of this [referring to thinking about biases] because the more we think about it, the more biases we identify and connect. So, having a way to map them in the process makes one think straighter and not get lost. For example, municipalities are responsible for funding primary care institutions. So, there is an unavoidable inequality in the distribution of resources; the municipalities with more money have better healthcare and better systems to manage the data. And there you go, another bias!”

The developers also observed the potential of the visualization, along with the guided prompts, in aiding them in managing the process of dealing with biases and their sources:

Developer A: “I mean, [identifying biases] is like one part of learning because when one really needs to be conscious about a problem, having different modes of analyzing that information is what helps. So, we had the interview and prompts; we got some insight from that, and then we also had visual support with the network maps, so having both things is what makes it better. It is not how information is being transferred but the fact that all these things are done simultaneously.”

Furthermore, the same developer noticed potential advantages in implementing this approach, both retrospectively (like they did) and also from the project’s outset, suggesting its application even before commencing data collection:

Developer A: “This could be implemented at two levels, in my opinion [pointing to the BNA maps]. One is the experimental design phase because asking all these questions and doing this sort of group analysis with the team makes the research better. The other one is to like, make things transparent, potential biases that my results have (that I perhaps cannot change), but that is worth publishing.”

Finally, the developer team mentioned a benefit that we had not thought about but that could be beneficial for them to track and defend the ethical issues or the more general ethical discussion of their work for article publication or conference calls:

Developer A: “I am not an expert in the area [referring to ethics], so for me, I like something that has utility so that I can add it to my paper, and if it also helps me make my research better, that’s good too. I can see how this can help you visualize, make decisions transparent, and socialize deficiencies in my project or experiment. So, it is a benefit for us [developers] but also for socializing our process with the community. In the end, this helps because making the choices about methods, data, and other decisions transparent helps to evaluate if something is good or bad.”

This made us consider how the BNA can be presented as a method for internal purposes (better research evaluation or ethical assessments) but also offer transparency elements for developers to socialize their decision-making process, particularly the ethical decisions, as part of their professional practice.

#### The Role of Collective Discussion in Encouraging Ethical Reflection

Likewise, they express an affinity for having a conversation. When asked what they thought about the method, Developer A stressed the benefits of thinking collectively as a team. They emphasized how this approach enabled collective consideration.

Developer A: “The best thing is the fact that someone external is asking us these questions and guiding us and that it is not heavily structured because problems were flowing and we could have a conversation, so we were able to go to the things we thought were more important naturally.”

Developer A: “There are a lot of benefits to implementing it [the approach] at the beginning and the end because I can check problems and maybe change the course of action. Even retrospectively, it helps you think about how to model stuff, like presenting it in a way that makes you think about these issues throughout.”

In the same spirit of a collective discussion, developer A said that the fact it was shown as a network helped because it is a better and more natural way to understand a problem.

Developer A: “Well, it feels more organic because that is how you should analyze a problem. If you analyze things part by part, it is easier, but you overlook the continuum—the interaction of the characteristics you are trying to systematize. Different aspects are not independent, so I think it helps because it helps you generalize the analysis, so you do not have a discreet analysis, but instead of the relation amongst those parts.”

This was particularly relevant to us, as this is part of the fundamental objective of a sociotechnical approach like the BNA, which reflects context. Given that the developer team’s own concerns and thought processes rather than predetermined assumptions or expectations drive it, mapping relationships between key elements is an approachable way to promote context awareness without overwhelming users.

## Conclusions and Future Work

AI ethics now require developers to contextualize their work more than ever, despite not always having ethical training. Based on our findings applying the BNA, we notice that facilitating ethical discussion is essential to establishing an understanding of ethical concepts for engineers lacking formal ethics training. Facilitation offers structure and ensures depth in discussions, enabling teams to effectively engage with the proposed method. The BNA provides a framework to help them visualize and understand the connections and nuances of AI biases, showing how addressing bias through a network leads to more transparent practices and decision-making. While this framework does not solve every ethical issue, it aims to help developers see how different ethical factors interact.

The proposed methodology, used for tracking and mapping bias-related elements and influences, demonstrated its utility for developers in the retrospective case study. Key aspects that make this sociotechnical approach valuable include:

- It is a people-based approach. The BNA acts as a link between technical aspects and societal concerns, emphasizing collaboration among developers. It encourages conversations, identifies factors that could lead to bias and takes into account outside limitations. It also involves experts who stimulate and guide these discussions. Although this requires significant human resources, the development team sees it as crucial. Their positive reaction to applying the BNA responded to the various elements it incorporates, like visual maps, discussions, and prompts, to enhance ethical awareness. Future work could explore the impact of applying the BNA several times; we expect that once familiarized, teams can be better prepared to lead autonomous discussions aligned with the BNA, allowing for a gradual transition that supports its integration into their AI development processes. To support that transition to autonomous use, alternative mechanisms could be developed to incorporate our conversational, prompt-based approach to trigger ethical discussions within AI teams.
- It makes ethical discussion accessible. A key present challenge is to increase AI ethical awareness among developers who may not have formal ethics training. The BNA was well-received for its user-friendliness. It helped developers reflect on their choices individually and as a team by incorporating parts of the AI process and specific prompts into their workflow. As a result, developers became active participants in ethical evaluations rather than just observers or passive checklist followers. This made it easier for them to understand and align with ethical guidelines and principles because they were personally connected to the issues, helping them see their relevance to their work.
- It can facilitate transparency. Developers found that using a visual bias network clarified their decision-making and the complex factors involved. This method allowed them to explain their decisions clearly, improving communication. This was seen as useful not just for academic papers but also in practical settings, like working with the Ministry of Health. This approach goes beyond simple checklists or steps; it reveals how different elements, including ethical principles, are interconnected and how developers are making connections amongst these elements to justify their decision-making. This aspect can enhance transparency and provide a path to mapping responsibilities within AI teams.

These findings, given the nature of our qualitative research paradigm, are not expected to be generalizable results. The focus is on providing a perspective on the drawbacks and advantages of using this network approach from the developer team’s perspective.

Additionally, the BNA’s application to a specific case allowed us to identify sources of biases that had pervasive, networked impacts on the project under study, such as professional biases and material limitations. Both had a significant influence on the project’s results and the emergence of additional biases in different steps of the AI process from data generation to selection of model performance metrics. However, they remain understudied in prior AI bias literature. While this invisibilization could be related to the isolationist approach we found in the bias literature, we notice that professional biases are barely even conceptualized as a bias present in AI, perhaps due to the lack of acknowledgement of AI developers that their work and training is not neutral and might be influenced by their own training. Thus, future research could further explore professional biases and material limitations in a broader variety of AI projects to assess their impacts and prevalence.

Building upon the pilot case study’s findings, we will design further case studies with other teams and projects to identify any tendencies regarding the general benefits of applying the approach, its limitations, the methodological improvements it can offer to the developers, and the influence of the approach on developers’ awareness of their ethical decision-making process. We intend to provide concrete guidance on how to apply the BNA to different types of projects and how to integrate it with existing ethical evaluations in future work. For now, we offer some preliminary guidance in the Annex.

## Appendix

### Preliminary Guidance on How to Apply the BNA

In our case study, we are aware that there could be certain biases in how the prompter questions were phrased or approached, so we proactively addressed potential biases by relying on the diverse background of our prompter team and adhering to a consistent methodology in how developers were guided in the discussion. These measures aimed to ground our findings in actual data and varied perspectives, ensuring the responses accurately represented participants' experiences. We suggest that other prompter teams do the same.

We break down the guidelines gathered from this pilot case study into three stages: the preliminary stage (preparation), the intervention stage (interaction and reflection), and the follow-up stage (evaluation and adjustment).

1. Preliminary stage

This stage involves gathering essential information about the AI project to gear up for the intervention phase.

- For the Developer Team:

Profile Identification: The team should clarify their experience, interdisciplinary work history, social impact project involvement, specializations, and any previous ethical evaluations. This step can occur pre-interview or during the intervention, aiming to recognize the team's collective and individual strengths and weaknesses.

Project Scope and Principles: Clearly articulate the AI project's objectives, problem, data usage, and intended application. It's crucial to also identify the guiding principles and discuss any considerations (or lack thereof) during the intervention.

- For the Prompter Team:

Project Acquaintance: Gain an in-depth understanding of the AI project's goals, stages, data sources, potential biases, and broader context. This knowledge is crucial for assessing societal impacts and guiding the development process.

Prompt Preparation: Develop prompts focusing on common biases and those specific to the project's context and team composition. The prompts should be relevant, open-ended, multi-dimensional, considerate of the team's disciplinary background, flexible, and avoid leading questions.

2. Intervention stage

This stage is the active engagement between the developers and prompters, employing the bias network methodology to scrutinize and map biases from a sociotechnical lens.

- For the prompters:

Semi-structured Interviews: Facilitate discussions to unearth relevant project elements and biases through guided interviews, encouraging developer dialogue and collaboration.

Network Map Illustration: Visualize connections between biases and principles using a color-coded, accessible AI pipeline structure. This map should be adaptable, highlighting relevant connections and serving as an interactive tool during sessions.

Development Documentation Encouragement: Urge developers to document their process, enhancing transparency and accountability. This documentation should reflect decisions, changes, and justifications, providing insights into the ethical underpinnings of technical choices.

3. Follow-up stage

This stage involves revisiting and integrating the intervention's findings into the developer team's design and development choices.

- For the Developer Team:

Should further queries or changes arise, the team is encouraged to re-engage with the prompters, possibly leading to additional interventions. Continuous documentation of emerging factors and decisions is vital for effective follow-ups.

**Timeline for Network Approach**

Based on the pilot study, estimated time investments are suggested for both teams, acknowledging that actual times may vary based on project specifics. The pilot case required approximately 8 h per prompter and about 3 h for the developer team. For new or early-stage projects, developers might anticipate more extended engagement and documentation as part of their ongoing development tasks, aiming for a continuous sociotechnical approach.

See Tables 1, 2 and 3.

**Table 1:** Literature review to build the standard pipeline for AI development

	Authors and date	Title	Pipeline stages
1	Akter et al. (2021)	Algorithmic bias in data-driven innovation in the age of AI	1. Data product conceptualization 2. Data acquisition and refinement 3. Data storage and retrieval 4. Data product distribution 5. Data product presentation 6. Market feedback
2	Baker & Hawn ac (2021)	Algorithmic bias in education	1. Data generation 2. Task definition 3. Data measurement 4. Model learning 5. Evaluation and post-processing 6. Model Deployment 7. Feedback and stakeholders
3	Barocas & Selbst (2016)	Big data's disparate impact	1. Defining target variable 2. Data training 3. Feature selection
4	Char et al. (2020)	Identifying ethical considerations for machine learning healthcare applications	1. Conception 2. Development 3. Calibration 4. Inspection 5. Initial implementation 6. Evaluation
5	Cramer et al. (2018)	Assessing and addressing algorithmic bias in practice	1. Input data 2. Algorithm and team decisions 3. Results
6	Danks & London (2017)	Algorithmic bias in autonomous systems	1. Training data 2. Algorithm design 3. Implementation and interpretation
7	Dobbe et al. (2018)	A broader view on bias in automated decision-making: reflecting on epistemology and dynamics	1. Design 2. Development 3. Implementation
8	Draude et al. (2019)	Situated algorithms: a socio-technical systemic approach to bias	1. Data stage 2. Algorithmic design 3. Implementation
9	United States Home Office (2016)	Big data: a report on algorithmic systems, opportunity, and civil rights	1. Input stage 2. Design of algorithmic systems and machine learning
10	Fazelpour & Danks (2021)	Algorithmic bias: senses, sources, solutions	1. Problem specificationTa 2. Data collection and pre-processing 3. Modelling and validation 4. Deployment
11	Feuerriegel et al. (2020)	Fair AI: challenges and opportunities	1. Data 2. Modelling 3. Inadequate implementation 4. Model learning: 5. Evaluation and post-processing 6. Model Deployment 7. Feedback from stakeholders
12	Johnson (2020)	Algorithmic bias: on the implicit biases of social technology	No explicit pipeline Mentions data collection, labeling, and model design stages
13	Kizilcec and Lee (2022)	Algorithmic fairness in education. In the ethics of artificial intelligence in education	1. Problem definition and data 2. Model learning 3. Action (output and implementation)
14	Mehrabi et al. (2021)	A survey on bias and fairness in machine learning	1. Data 2. Algorithm 3. User interaction
15	Mitchell et al. (2021)	Algorithmic fairness: choices, assumptions, and definitions	1.Problem definition 2. Data 3. Model design 4. Evaluation
16	Olteanu et al. (2019)	Social data: biases, methodological pitfalls, and ethical boundaries	1. Research design 2. Data collecting 3. Processing 4. Analysis 5. Evaluation
17	Parikh et al. (2019)	Addressing bias in artificial intelligence in health care	1. Data collection 2. Model design and implementation 3. Interpretation
18	Paullada et al. (2021)	Data and its (dis)contents: a survey of dataset development and use in machine learning research	1. Data collection 2. Data annotation 3. Documentation 4. Benchmarking 5. Data reuse
19	Richardson & Gilbert (2021)	A framework for fairness: a systematic review of existing fair AI solutions	1. Data collection 2. Data processing 3. Implementation and interpretation
20	Roselli et al. (2019)	Managing bias in AI	1. Goal definition 2. Data stage 3. Implementation
21	Rovatsos et al. (2019)	Landscape summary: bias in algorithmic decision-making	1. Input stage 2. Algorithm stage 3. Implementation and evaluation 4. Interpretation
22	Smith & Rustagi (2020)	Mitigating bias in artificial intelligence. an equity fluent leadership playbook	1. Data collection/ labelling 2. Algorithmic design and evaluation 3. Implementation and interpretation
23	Suresh & Guttag (2021)	A framework for understanding sources of harm throughout the machine learning life cycle	1. Data collection 2. Data preparation 3. Model development 4. Model evaluation 5. Model post-processing 6. Model deployment
24	Sangokoya (2020)	Algorithmic accountability – Applying the concept to different country contexts	1. Input data 2. Processing and weighting of data 3. Implementation 4. Feedback
25	Srinivasan & Chander (2021)	Biases in AI systems	1. Data collection, labelling and pre-processing 2. Problem formulation 3. Algorithm and analysis 4. Testing and validation
26	Završnik (2021)	Algorithmic justice: Algorithms and big data in criminal justice settings	1. Database building 2. Algorithm design 3. Implementation 4. Interpretation 5. Feedback

**Table 2:** Biases at different stages of the AI pipeline, as often mentioned in the literature

	Societal biases	Cognitive biases	Technical biases
Problem formulation	Institutional (Ntoutsi et al., 2020) and historical (Suresh & Guttag, 2021)	Influence of human bias (Smith & Rustagi, 2020)	
Data collection/generation	Historical (Char et al., 2020, Adebe et al., 2021, Mehrabi et al., 2021)	Social desirability (Mehrabi et al., 2021)	Sampling (Baker & Hawn, 2021; Cramer et al., 2018; Suresh & Guttag, 2021), Representation (Mehrabi et al., 2021; Suresh & Guttag, 2021), Aggregation (Mehrabi et al., 2021), Measurement (Dobbe et al., 2018; Mehrabi et al., 2021), Selection (Akter et al., 2021), Self-selection (Cramer et al., 2018)
Preprocessing/labeling		Labeling (Char et al., 2020; Dobbe et al., 2018; Mehrabi et al., 2021; Smith & Rustagi, 2020; Srinivasan & Chander, 2021): Prejudiced labelling choices (Barocas & Selbst, 2016)	
Model development/calibration		Confirmation (Srinivasan & Chander, 2021) and automation (Dobbe et al., 2018)	Aggregation (Baker & Hawn, 2021; Olteanu et al., 2019), modeling (Barocas & Selbst, 2016; Dobbe et al., 2018), algorithmic processing bias, biased estimators (Danks & London, 2017), optimization (Dobbe et al., 2018; Fazelpour & Danks, 2021), and skewed processing (Rovatsos, Mittelstadt et al., 2019), feedback loops (Akter et al., 2021), evaluation (Fazelpour & Danks, 2021; Olteanu et al., 2019; Smith & Rustagi, 2020)
Implementation			Emerging (Draude et al., 2019), feedback collects biased data (Feuerriegel et al., 2020)
Interpretation		Confirmation bias (Srinivasan & Chander, 2021), Recall bias (Srinivasan & Chander, 2021), Previous beliefs interference (Srinivasan & Chander, 2021) User knowledge bias (Richardson & Gilbert, 2021; Draude et al., 2019) Gamification bias (Dobbe et al., 2018; Richardson & Gilbert, 2021)

**Table 3:** Sources of biases at different stages of the AI pipeline, as often mentioned in the literature

	Other sources of biased AI
Problem formulation	Lack of clear goals and criteria (Roselli, et al., 2019), Mismatch between problem and population/context (Mitchell et al., 2021), Mismatch between problem and variables (Barocas & Selbst, 2016) Lack of diverse perspectives when formulating problem (Baker & Hawn, 2021)
Data collection/generation	Mismatch between human construct and data (Richardson & Gilbert, 2021), Variable selection (Fazelpour & Danks, 2021), Limitations associated with data (Mehrabi et al., 2021; Olteanu et al., 2019), Issues with variable granularity (Barocas & Selbst, 2016; Feuerriegel et al., 2020; Kizilcec & Lee, 2022; Mehrabi et al., 2021), Poor data or training data quality (Rovatsos, et al., 2019; Sangokoya, 2017; United States Home Office, 2016), Incomplete data (United States Home Office, 2016), missing data (Parikh et al., 2019), unseen data (Roselli, Matthews & Talagala, 2019), omitting sensible data (Barocas & Selbst, 2016; Mehrabi et al., 2021; Ntoutsi et al., 2020), inaccurate data (Roselli et al., 2019), outdated data (United States Home Office, 2016), manipulated data (Roselli et al., 2019), presence of outliers (Parikh, et al., 2019), Problems with data format (Ntoutsi et al., 2020), Limitations related to the type of data (Mehrabi et al., 2021; Olteanu et al., 2019)
Preprocessing/labelling	Poor data notation (Paullada et al., 2021)
Model development/calibration	Spurious correlations (Parikk et al., 2019), Proxy variables (Feuerriegel, et al., 2020) and surrogate data (Mitchell et al., 2021; Roselli et al., 2019) Differential use of variables (Danks & London, 2017) Poorly weighted data (Sangokoya, 2020) Use of illegal variables (Danks & London, 2017) Symmetrical considerations for all individuals (Chouldechova & Roth, 2020) Insufficient procedures and computer tools’ limitations (Richardson & Gilbert, 2021) Assuming that technical decisions do not affect outcomes (Hooker, 2021) Assuming simultaneous evaluations (Mitchell et al., 2021) Reductive representation (Barocas & Selbst, 2016) Inadequate performance metrics (Suresh & Guttag, 2021) Inappropriate benchmarks (Mehrabi et al., 2021; Paullada et al., 2021; Srinivasan & Chander, 2021; Suresh & Guttag, 2021)and benchmarks used in inadequate context (Paullada et al., 2021) Inadequate fairness evaluation (Mitchell et al., 2021) or criteria (Fazelpour & Danks, 2021), tradeoff between fairness and accuracy (Char et al., 2020)
Implementation	Lack of preventive actions and monitoring (Char et al., 2020; Fazelpour & Danks, 2021) Automatic implementation (Akter et al., 2021) Arbitrary utilization of the model (Richardson & Gilbert, 2021) Mismatch between intended design and use (Draude et al., 2019)
Interpretation	Assuming causation (Draude et al., 2019)
